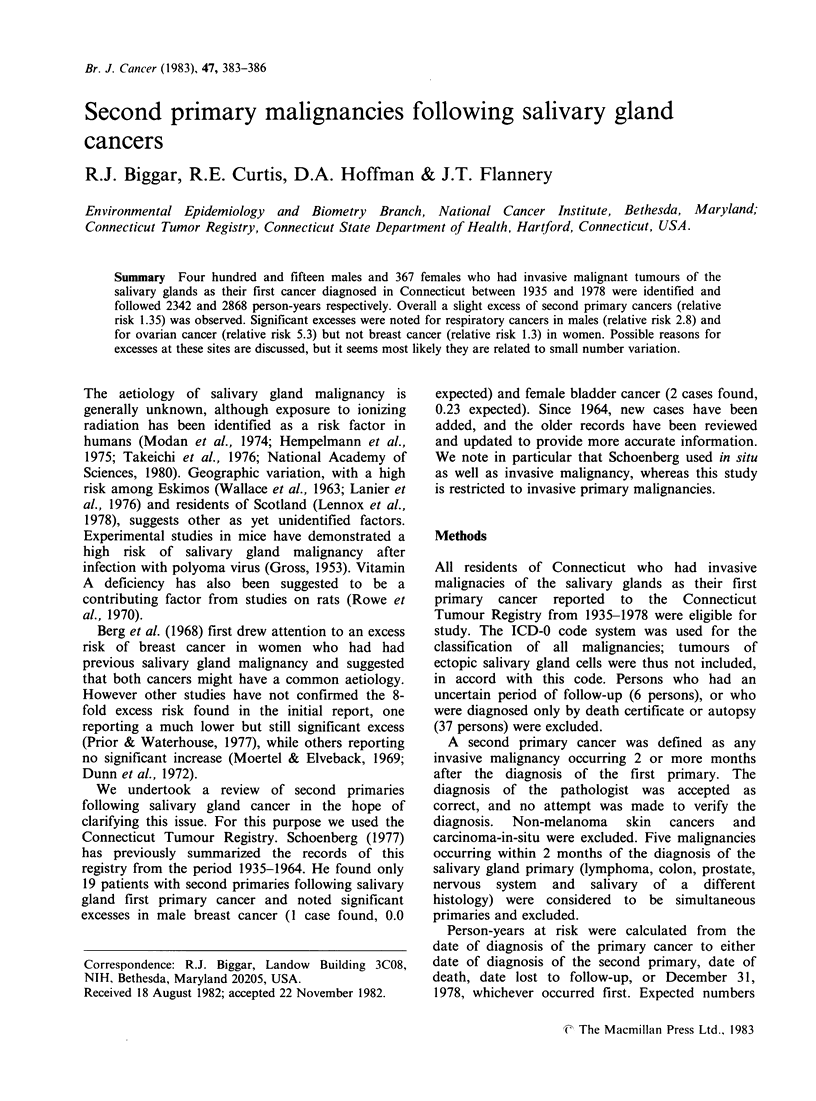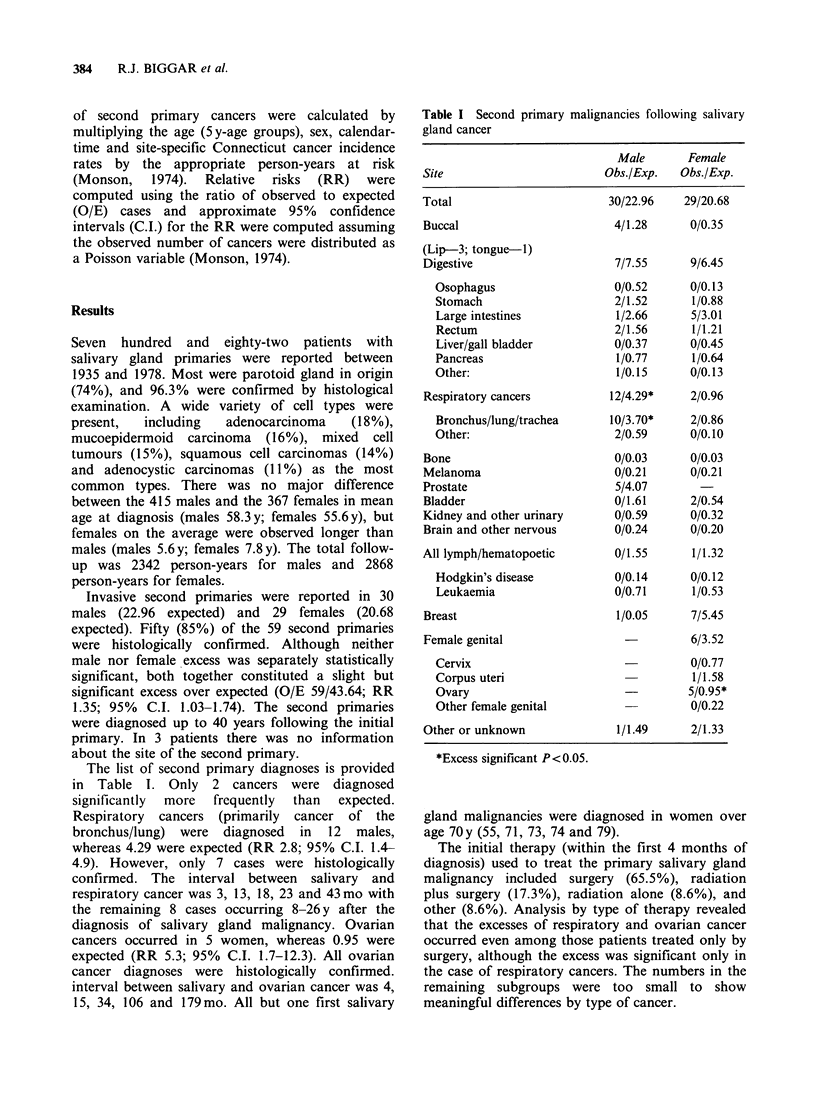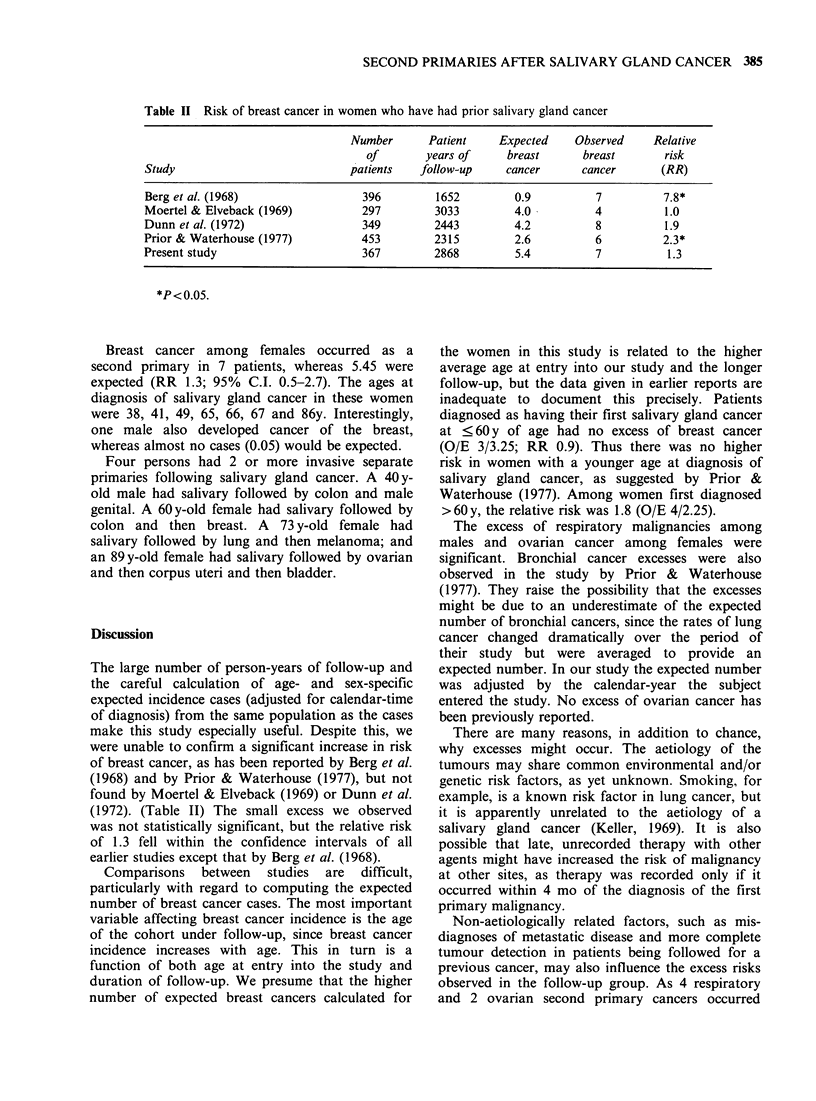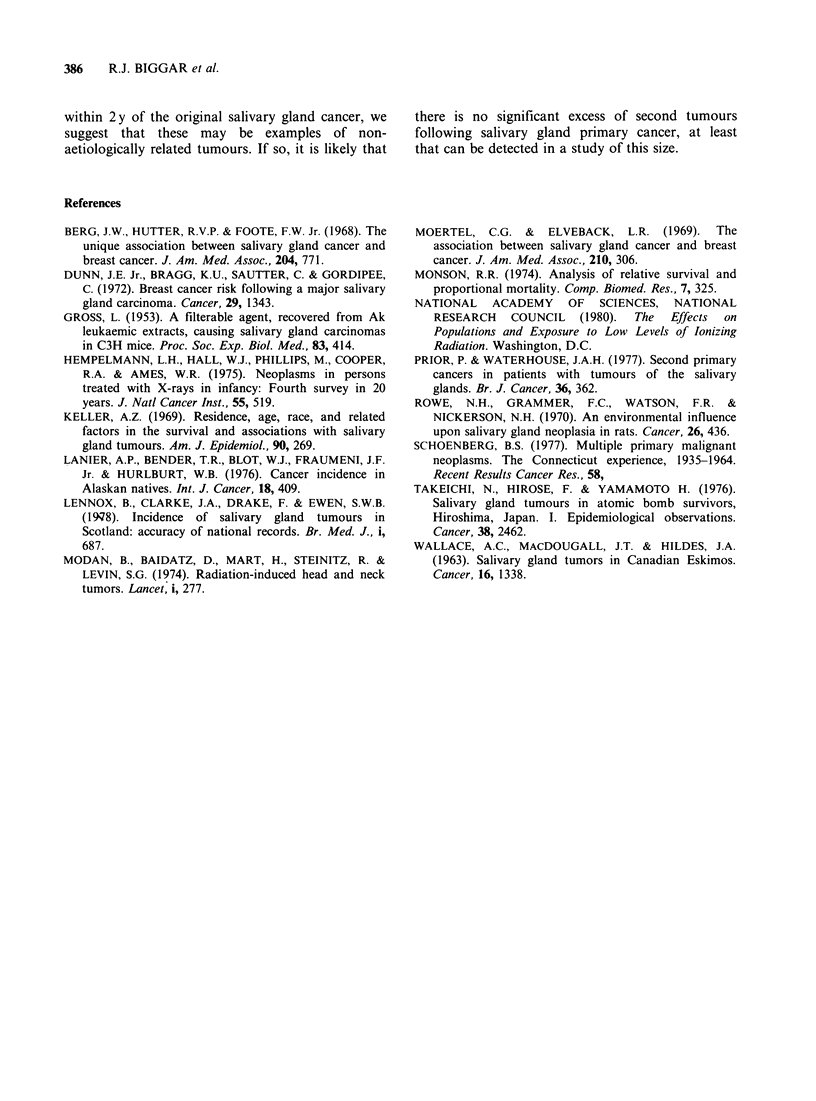# Second primary malignancies following salivary gland cancers.

**DOI:** 10.1038/bjc.1983.57

**Published:** 1983-03

**Authors:** R. J. Biggar, R. E. Curtis, D. A. Hoffman, J. T. Flannery

## Abstract

Four hundred and fifteen males and 367 females who had invasive malignant tumours of the salivary glands as their first cancer diagnosed in Connecticut between 1935 and 1978 were identified and followed 2342 and 2868 person-years respectively. Overall a slight excess of second primary cancers (relative risk 1.35) was observed. Significant excesses were noted for respiratory cancers in males (relative risk 2.8) and for ovarian cancer (relative risk 5.3) but not breast cancer (relative risk 1.3) in women. Possible reasons for excesses at these sites are discussed, but it seems most likely they are related to small number variation.


Br. J. Cancer (1983), **47**, 383–386

### Second primary malignancies following salivary gland cancers

R.J. Biggar, R.E. Curtis, D.A. Hoffman & J.T. Flannery

*Environmental Epidemiology and Biometry Branch, National Cancer Institute, Bethesda, Maryland; Connecticut Tumor Registry, Connecticut State Department of Health, Hartford, Connecticut, USA.*

**Summary** Four hundred and fifteen males and 367 females who had invasive malignant tumours of the salivary glands as their first cancer diagnosed in Connecticut between 1935 and 1978 were identified and followed 2342 and 2868 person-years respectively. Overall a slight excess of second primary cancers (relative risk 1.35) was observed. Significant excesses were noted for respiratory cancers in males (relative risk 2.8) and for ovarian cancer (relative risk 5.3) but not breast cancer (relative risk 1.3) in women. Possible reasons for excesses at these sites are discussed, but it seems most likely they are related to small number variation.

The aetiology of salivary gland malignancy is generally unknown, although exposure to ionizing radiation has been identified as a risk factor in humans (Modan *et al.*, 1974; Hempelmann *et al.*, 1975; Takeichi *et al.*, 1976; National Academy of Sciences, 1980). Geographic variation, with a high risk among Eskimos (Wallace *et al.*, 1963; Lanier *et al.*, 1976) and residents of Scotland (Lennox *et al.*, 1978), suggests other as yet unidentified factors. Experimental studies in mice have demonstrated a high risk of salivary gland malignancy after infection with polyoma virus (Gross, 1953). Vitamin A deficiency has also been suggested to be a contributing factor from studies on rats (Rowe *et al.*, 1970).

Berg *et al.* (1968) first drew attention to an excess risk of breast cancer in women who had had previous salivary gland malignancy and suggested that both cancers might have a common aetiology. However other studies have not confirmed the 8-fold excess risk found in the initial report, one reporting a much lower but still significant excess (Prior & Waterhouse, 1977), while others reporting no significant increase (Moertel & Elveback, 1969; Dunn *et al.*, 1972).

We undertook a review of second primaries following salivary gland cancer in the hope of clarifying this issue. For this purpose we used the Connecticut Tumour Registry. Schoenberg (1977) has previously summarized the records of this registry from the period 1935–1964. He found only 19 patients with second primaries following salivary gland first primary cancer and noted significant excesses in male breast cancer (1 case found, 0.0 expected) and female bladder cancer (2 cases found, 0.23 expected). Since 1964, new cases have been added, and the older records have been reviewed and updated to provide more accurate information. We note in particular that Schoenberg used *in situ* as well as invasive malignancy, whereas this study is restricted to invasive primary malignancies.

Correspondence: R.J. Biggar, Landow Building 3C08, NIH, Bethesda, Maryland 20205, USA.
Received 18 August 1982; accepted 22 November 1982.

### Methods

All residents of Connecticut who had invasive malignacies of the salivary glands as their first primary cancer reported to the Connecticut Tumour Registry from 1935–1978 were eligible for study. The ICD-0 code system was used for the classification of all malignancies; tumours of ectopic salivary gland cells were thus not included, in accord with this code. Persons who had an uncertain period of follow-up (6 persons), or who were diagnosed only by death certificate or autopsy (37 persons) were excluded.

A second primary cancer was defined as any invasive malignancy occurring 2 or more months after the diagnosis of the first primary. The diagnosis of the pathologist was accepted as correct, and no attempt was made to verify the diagnosis. Non-melanoma skin cancers and carcinoma-in-situ were excluded. Five malignancies occurring within 2 months of the diagnosis of the salivary gland primary (lymphoma, colon, prostate, nervous system and salivary of a different histology) were considered to be simultaneous primaries and excluded.

Person-years at risk were calculated from the date of diagnosis of the primary cancer to either date of diagnosis of the second primary, date of death, date lost to follow-up, or December 31, 1978, whichever occurred first. Expected numbers

© The Macmillan Press Ltd., 1983

of second primary cancers were calculated by multiplying the age (5 y-age groups), sex, calendar-time and site-specific Connecticut cancer incidence rates by the appropriate person-years at risk (Monson, 1974). Relative risks (RR) were computed using the ratio of observed to expected (O/E) cases and approximate 95% confidence intervals (C.I.) for the RR were computed assuming the observed number of cancers were distributed as a Poisson variable (Monson, 1974).

### Results

Seven hundred and eighty-two patients with salivary gland primaries were reported between 1935 and 1978. Most were parotoid gland in origin (74%), and 96.3% were confirmed by histological examination. A wide variety of cell types were present, including adenocarcinoma (18%), mucoepidermoid carcinoma (16%), mixed cell tumours (15%), squamous cell carcinomas (14%) and adenocystic carcinomas (11%) as the most common types. There was no major difference between the 415 males and the 367 females in mean age at diagnosis (males 58.3 y; females 55.6 y), but females on the average were observed longer than males (males 5.6 y; females 7.8 y). The total follow-up was 2342 person-years for males and 2868 person-years for females.

Invasive second primaries were reported in 30 males (22.96 expected) and 29 females (20.68 expected). Fifty (85%) of the 59 second primaries were histologically confirmed. Although neither male nor female excess was separately statistically significant, both together constituted a slight but significant excess over expected (O/E 59/43.64; RR 1.35; 95% C.I. 1.03–1.74). The second primaries were diagnosed up to 40 years following the initial primary. In 3 patients there was no information about the site of the second primary.

The list of second primary diagnoses is provided in Table I. Only 2 cancers were diagnosed significantly more frequently than expected. Respiratory cancers (primarily cancer of the bronchus/lung) were diagnosed in 12 males, whereas 4.29 were expected (RR 2.8; 95% C.I. 1.4–4.9). However, only 7 cases were histologically confirmed. The interval between salivary and respiratory cancer was 3, 13, 18, 23 and 43 mo with the remaining 8 cases occurring 8–26 y after the diagnosis of salivary gland malignancy. Ovarian cancers occurred in 5 women, whereas 0.95 were expected (RR 5.3; 95% C.I. 1.7–12.3). All ovarian cancer diagnoses were histologically confirmed. interval between salivary and ovarian cancer was 4, 15, 34, 106 and 179 mo. All but one first salivary gland malignancies were diagnosed in women over age 70 y (55, 71, 73, 74 and 79).

The initial therapy (within the first 4 months of diagnosis) used to treat the primary salivary gland malignancy included surgery (65.5%), radiation plus surgery (17.3%), radiation alone (8.6%), and other (8.6%). Analysis by type of therapy revealed that the excesses of respiratory and ovarian cancer occurred even among those patients treated only by surgery, although the excess was significant only in the case of respiratory cancers. The numbers in the remaining subgroups were too small to show meaningful differences by type of cancer.

**Table I** Second primary malignancies following salivary gland cancer

| *Site* | *Male Obs./Exp.* | *Female Obs./Exp.* |
|---|---|---|
| Total | 30/22.96 | 29/20.68 |
| Buccal | 4/1.28 | 0/0.35 |
| (Lip—3; tongue—1) | | |
| Digestive | 7/7.55 | 9/6.45 |
| Osophagus | 0/0.52 | 0/0.13 |
| Stomach | 2/1.52 | 1/0.88 |
| Large intestines | 1/2.66 | 5/3.01 |
| Rectum | 2/1.56 | 1/1.21 |
| Liver/gall bladder | 0/0.37 | 0/0.45 |
| Pancreas | 1/0.77 | 1/0.64 |
| Other: | 1/0.15 | 0/0.13 |
| Respiratory cancers | 12/4.29* | 2/0.96 |
| Bronchus/lung/trachea | 10/3.70* | 2/0.86 |
| Other: | 2/0.59 | 0/0.10 |
| Bone | 0/0.03 | 0/0.03 |
| Melanoma | 0/0.21 | 0/0.21 |
| Prostate | 5/4.07 | — |
| Bladder | 0/1.61 | 2/0.54 |
| Kidney and other urinary | 0/0.59 | 0/0.32 |
| Brain and other nervous | 0/0.24 | 0/0.20 |
| All lymph/hematopoetic | 0/1.55 | 1/1.32 |
| Hodgkin's disease | 0/0.14 | 0/0.12 |
| Leukaemia | 0/0.71 | 1/0.53 |
| Breast | 1/0.05 | 7/5.45 |
| Female genital | — | 6/3.52 |
| Cervix | — | 0/0.77 |
| Corpus uteri | — | 1/1.58 |
| Ovary | — | 5/0.95* |
| Other female genital | — | 0/0.22 |
| Other or unknown | 1/1.49 | 2/1.33 |

*Excess significant $P<0.05$.

**Table II** Risk of breast cancer in women who have had prior salivary gland cancer

| *Study* | *Number of patients* | *Patient years of follow-up* | *Expected breast cancer* | *Observed breast cancer* | *Relative risk (RR)* |
|---|---|---|---|---|---|
| Berg *et al.* (1968) | 396 | 1652 | 0.9 | 7 | 7.8* |
| Moertel & Elveback (1969) | 297 | 3033 | 4.0 | 4 | 1.0 |
| Dunn *et al.* (1972) | 349 | 2443 | 4.2 | 8 | 1.9 |
| Prior & Waterhouse (1977) | 453 | 2315 | 2.6 | 6 | 2.3* |
| Present study | 367 | 2868 | 5.4 | 7 | 1.3 |

*$P<0.05$.

Breast cancer among females occurred as a second primary in 7 patients, whereas 5.45 were expected (RR 1.3; 95% C.I. 0.5–2.7). The ages at diagnosis of salivary gland cancer in these women were 38, 41, 49, 65, 66, 67 and 86y. Interestingly, one male also developed cancer of the breast, whereas almost no cases (0.05) would be expected.

Four persons had 2 or more invasive separate primaries following salivary gland cancer. A 40 y-old male had salivary followed by colon and male genital. A 60 y-old female had salivary followed by colon and then breast. A 73 y-old female had salivary followed by lung and then melanoma; and an 89 y-old female had salivary followed by ovarian and then corpus uteri and then bladder.

### Discussion

The large number of person-years of follow-up and the careful calculation of age- and sex-specific expected incidence cases (adjusted for calendar-time of diagnosis) from the same population as the cases make this study especially useful. Despite this, we were unable to confirm a significant increase in risk of breast cancer, as has been reported by Berg *et al.* (1968) and by Prior & Waterhouse (1977), but not found by Moertel & Elveback (1969) or Dunn *et al.* (1972). (Table II) The small excess we observed was not statistically significant, but the relative risk of 1.3 fell within the confidence intervals of all earlier studies except that by Berg *et al.* (1968).

Comparisons between studies are difficult, particularly with regard to computing the expected number of breast cancer cases. The most important variable affecting breast cancer incidence is the age of the cohort under follow-up, since breast cancer incidence increases with age. This in turn is a function of both age at entry into the study and duration of follow-up. We presume that the higher number of expected breast cancers calculated for the women in this study is related to the higher average age at entry into our study and the longer follow-up, but the data given in earlier reports are inadequate to document this precisely. Patients diagnosed as having their first salivary gland cancer at ≤60 y of age had no excess of breast cancer (O/E 3/3.25; RR 0.9). Thus there was no higher risk in women with a younger age at diagnosis of salivary gland cancer, as suggested by Prior & Waterhouse (1977). Among women first diagnosed >60 y, the relative risk was 1.8 (O/E 4/2.25).

The excess of respiratory malignancies among males and ovarian cancer among females were significant. Bronchial cancer excesses were also observed in the study by Prior & Waterhouse (1977). They raise the possibility that the excesses might be due to an underestimate of the expected number of bronchial cancers, since the rates of lung cancer changed dramatically over the period of their study but were averaged to provide an expected number. In our study the expected number was adjusted by the calendar-year the subject entered the study. No excess of ovarian cancer has been previously reported.

There are many reasons, in addition to chance, why excesses might occur. The aetiology of the tumours may share common environmental and/or genetic risk factors, as yet unknown. Smoking, for example, is a known risk factor in lung cancer, but it is apparently unrelated to the aetiology of a salivary gland cancer (Keller, 1969). It is also possible that late, unrecorded therapy with other agents might have increased the risk of malignancy at other sites, as therapy was recorded only if it occurred within 4 mo of the diagnosis of the first primary malignancy.

Non-aetiologically related factors, such as misdiagnoses of metastatic disease and more complete tumour detection in patients being followed for a previous cancer, may also influence the excess risks observed in the follow-up group. As 4 respiratory and 2 ovarian second primary cancers occurred within 2 y of the original salivary gland cancer, we suggest that these may be examples of non-aetiologically related tumours. If so, it is likely that there is no significant excess of second tumours following salivary gland primary cancer, at least that can be detected in a study of this size.

### References

BERG, J.W., HUTTER, R.V.P. & FOOTE, F.W. Jr. (1968). The unique association between salivary gland cancer and breast cancer. *J. Am. Med. Assoc.*, **204,** 771.

DUNN, J.E. Jr., BRAGG, K.U., SAUTTER, C. & GORDIPEE, C. (1972). Breast cancer risk following a major salivary gland carcinoma. *Cancer,* **29,** 1343.

GROSS, L. (1953). A filterable agent, recovered from Ak leukaemic extracts, causing salivary gland carcinomas in C3H mice. *Proc. Soc. Exp. Biol. Med.,* **83,** 414.

HEMPELMANN, L.H., HALL, W.J., PHILLIPS, M., COOPER, R.A. & AMES, W.R. (1975). Neoplasms in persons treated with X-rays in infancy: Fourth survey in 20 years. *J. Natl Cancer Inst.,* **55,** 519.

KELLER, A.Z. (1969). Residence, age, race, and related factors in the survival and associations with salivary gland tumours. *Am. J. Epidemiol.,* **90,** 269.

LANIER, A.P., BENDER, T.R., BLOT, W.J., FRAUMENI, J.F. Jr. & HURLBURT, W.B. (1976). Cancer incidence in Alaskan natives. *Int. J. Cancer,* **18,** 409.

LENNOX, B., CLARKE, J.A., DRAKE, F. & EWEN, S.W.B. (1978). Incidence of salivary gland tumours in Scotland: accuracy of national records. *Br. Med. J.,* **i,** 687.

MODAN, B., BAIDATZ, D., MART, H., STEINITZ, R. & LEVIN, S.G. (1974). Radiation-induced head and neck tumors. *Lancet,* **i,** 277.

MOERTEL, C.G. & ELVEBACK, L.R. (1969). The association between salivary gland cancer and breast cancer. *J. Am. Med. Assoc.,* **210,** 306.

MONSON, R.R. (1974). Analysis of relative survival and proportional mortality. *Comp. Biomed. Res.,* **7,** 325.

NATIONAL ACADEMY OF SCIENCES, NATIONAL RESEARCH COUNCIL (1980). *The Effects on Populations and Exposure to Low Levels of Ionizing Radiation.* Washington, D.C.

PRIOR, P. & WATERHOUSE, J.A.H. (1977). Second primary cancers in patients with tumours of the salivary glands. *Br. J. Cancer,* **36,** 362.

ROWE, N.H., GRAMMER, F.C., WATSON, F.R. & NICKERSON, N.H. (1970). An environmental influence upon salivary gland neoplasia in rats. *Cancer,* **26,** 436.

SCHOENBERG, B.S. (1977). Multiple primary malignant neoplasms. The Connecticut experience, 1935–1964. *Recent Results Cancer Res.,* **58,**

TAKEICHI, N., HIROSE, F. & YAMAMOTO H. (1976). Salivary gland tumours in atomic bomb survivors, Hiroshima, Japan. I. Epidemiological observations. *Cancer,* **38,** 2462.

WALLACE, A.C., MACDOUGALL, J.T. & HILDES, J.A. (1963). Salivary gland tumors in Canadian Eskimos. *Cancer,* **16,** 1338.